# Health, wellbeing and nutritional status of older people living in UK care homes: an exploratory evaluation of changes in food and drink provision

**DOI:** 10.1186/1471-2318-10-28

**Published:** 2010-05-27

**Authors:** Andrea Kenkmann, Gill M Price, Joanne Bolton, Lee Hooper

**Affiliations:** 1 School of Medicine, Health Policy and Practice, University of East Anglia, Norwich NR4 7TJ, UK; 2 St Nicholas House, Dereham, Norfolk NR19 1BG, UK

## Abstract

**Background:**

Food and drink are important determinants of physical and social health in care home residents. This study explored whether a pragmatic methodology including routinely collected data was feasible in UK care homes, to describe the health, wellbeing and nutritional status of care home residents and assess effects of changed provision of food and drink at three care homes on residents' falls (primary outcome), anaemia, weight, dehydration, cognitive status, depression, lipids and satisfaction with food and drink provision.

**Methods:**

We measured health, wellbeing and nutritional status of 120 of 213 residents of six care homes in Norfolk, UK. An intervention comprising improved dining atmosphere, greater food choice, extended restaurant hours, and readily available snacks and drinks machines was implemented in three care homes. Three control homes maintained their previous system. Outcomes were assessed in the year before and the year after the changes.

**Results:**

Use of routinely collected data was partially successful, but loss to follow up and levels of missing data were high, limiting power to identify trends in the data.

This was a frail older population (mean age 87, 71% female) with multiple varied health problems. During the first year 60% of residents had one or more falls, 40% a wound care visit, and 40% a urinary tract infection. 45% were on diuretics, 24% antidepressants, and 43% on psychotropic medication.

There was a slight increase in falls from year 1 to year 2 in the intervention homes, and a much bigger increase in control homes, leading to a statistically non-significant 24% relative reduction in residents' rate of falls in intervention homes compared with control homes (adjusted rate ratio 0.76, 95% CI 0.57 to 1.02, p = 0.06).

**Conclusions:**

Care home residents are frail and experience multiple health risks. This intervention to improve food and drink provision was well received by residents, but effects on health indicators (despite the relative reduction in falls rate) were inconclusive, partly due to problems with routine data collection and loss to follow up. Further research with more homes is needed to understand which, if any, components of the intervention may be successful.

**Trial registration:**

Trial registration: ISRCTN86057119.

## Background

Four percent of older people in the UK live in a care home or long-stay hospital, rising to 21% of those aged at least 85[1]. Fifteen percent of the 486,000 places offered are run by Local Authorities (local government) or the National Health Service (NHS)[2]. Malnutrition is common in older people living in the community [3], in those admitted to care homes [4] and in care homes [5-7]. The importance of tackling malnutrition in older people in residential care has been nationally recognised [8,9] and a UK joint action plan has encouraged local authorities to "champion good nutritional care" in local homes by seeking and acting on feedback from service users on nutritional issues and quality of meals provided[8].

The effects of malnutrition on health and wellbeing in older people are serious. The UK National Institute for Health and Clinical Excellence reports that tackling malnutrition in hospital can reduce complications and deaths[10]. The effects of malnutrition and dehydration are likely to include increased falls, vulnerability to infection, loss of energy and mobility, poor wound healing, confusion and ultimately an increased risk of mortality. In nursing homes the presence of low body mass index (BMI) is associated with lower quality of life[11]. Food and drink also have a social importance over and above their health effects, providing comfort and stimulation. Food and drink are shared with family and friends or when there is reason to celebrate. Meals may be the highlight of the day for those in residential care[12-15].

The reasons for poor food and drink intake in older people are varied, and can include disease, loss of thirst and/or appetite, loss of taste or smell (which may occur with age or as a result of medication), problems with coordination, poor dentition, metabolic processes, swallowing difficulties (for example, after a stroke), psychological factors such as depression, anxiety or confusion and dementia[16-18]. The institutional setting, which may have rigid routines, low staffing levels and un-homely environment may exacerbate existing problems, by minimising the positive aspects of enjoyment and social contact[19]. Similarly, a narrow range of food choices is associated with poorer nutritional status[20].

For all these reasons Norfolk County Council wanted to improve food provision in the care homes they manage. The changed provision was planned as part of local service development and aimed to improve health and wellbeing of a frail older population at three care homes compared with three control homes (the new service was implemented in the control homes shortly after the final study assessments). This study set out to assess the health, wellbeing and nutritional status of a population of older people living in UK residential care, and to assess the feasibility of measuring the effects of a change in provision of food and drink in this context. The rate of residents' falls (primary outcome), anaemia, weight, dehydration, cognitive status, depression, serum lipids and satisfaction with food and drink provision were assessed.

## Methods

A change in provision of food and drink was developed and piloted in one Norfolk County Council run care home, with substantial investment in staff time, décor and equipment. The changes in this first home were judged very successful, endorsed by both staff and residents, and there was a suggestion of a reduction in numbers of residents' falls (30% fewer falls in the 3 months after the change compared with the 3 months before), reduction in falls needing assessment at the local accident and emergency department, better physical and mental state for several residents who had struggled to eat enough previously, increased interest in food, more socialising during meal times, and more relatives eating meals at the home.

For this reason it was decided to explore how we might address the effects of such changes more rigorously. We aimed to develop and assess a pragmatic methodology based on planned service development and maximising use of routine data collection while employing a controlled design and some additional measured outcomes. We set out to trial these changes in food and drink provision more formally in 3 further homes. As the 3 homes had already been chosen, the study design incorporated an additional 3 matched homes (matched by resident population and home size) as controls. We operated a pragmatic assessment using a mixture of pre-change and post-change data collected through questionnaires, interviews, routine data collected by the homes and blood tests. One intervention home and the matched control home specialised in dementia care as care homes in Norfolk were on the cusp of becoming mixed homes with some residents with dementia, enabling the appropriateness of changes across populations with differing levels of cognition to be addressed. Ethical approval for the study was obtained from the University of East Anglia, Faculty of Health, Ethics Committee. The trial was registered as ISRCTN86057119 (see http://www.controlled-trials.com/ISRCTN86057119).

**Figure 1:**
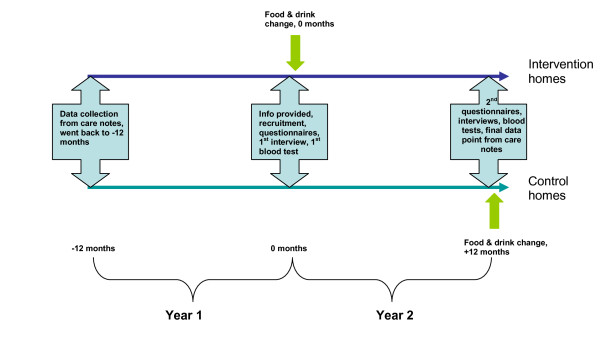
**Study time flow**. Study flow for intervention and control homes.

For a pictorial description of the study chronological flow see figure 1. During the spring and summer of 2006, a few weeks before the change in food and drink was due to take place in the intervention home, before baseline data collection or requests that residents take part in the study, residents, relatives and staff in each intervention home and its paired control were informed about the changes at open meetings (relatives were sent written invitations). All were told that the aim of the changes was to improve residents experience of eating and drinking in their homes, that there was a suggestion that this may impact positively on residents health (but that this was not clear) and that the changes were going to occur in some homes imminently, and in others a year later (with specific information provided on the home that was being addressed). There was concern among relatives that the changes were going to result in higher charges or were a cover for reduced meal quality or reduced care, and these concerns were directly addressed by Norfolk County Council managers at each home.

All residents, staff and relatives in control and intervention homes were given questionnaires (relatives were sent them in the post with stamped addressed envelopes, residents and staff were provided a box in which to post their completed forms) about current food and drink. During this same period residents were informed about the study and asked whether they would like to participate in any or all of the following: allowing researchers access to their routinely collected data (from a variety of sources, collectively referred to as 'care notes'); participating in an interview (to collect demographic data, mid-upper arm circumference, grip strength, hydration status, depression, anxiety and cognitive status); and/or providing a fasting blood sample.

Where interested, residents were asked to provide written informed consent. Home managers were approached to check whether any residents would be unable to provide informed consent due to impaired cognition - for such residents relatives were asked for informed assent (blood tests were not requested of such residents or their relatives). Where a relative provided informed assent an interview was only undertaken where the resident appeared happy and relaxed during the process - where this was not the case the interview was not attempted or immediately terminated. Ethically this system provided a double check from relatives that participants were likely to have approved of participation in the study when at full cognitive ability, and also appeared happy to participate on a moment-by-moment basis. As some residents were forgetful the study, its purpose and process were re-described at each meeting to every participant and all were offered chances to participate or not at each planned contact. While most residents who had provided consent were happy to participate in the interviews, several declined blood tests on the mornings that these were scheduled, due to feeling tired or unwell.

Care note (routine) data were collected in the intervention and control home pair for the year preceding the change at the intervention home (first year, much of this time prior to enrolment in the study), and for the year following the changeover (second year), see figure 1. This included whether the resident had fallen, number of falls, medications used, mobility, weights, height, major illnesses, contact with health care professionals and hospital admissions. Interviews were held just before the change over (first year), and one year later (second year).

Interviews included assessment of mid-upper arm circumference,[21,22] grip strength (measured by a battery powered Takei TKK5401 Grip D LED-display dynamometer, best of 3 measures on each side with arm held vertically downward at side of the chair), assessment of dehydration (visual assessment of the tongue by a trained district nurse), cognitive functioning (Mini-Mental State Exam [23], MMSE with scores from 0 to 30, higher scores representing better cognitive function), depression and anxiety (Hospital Anxiety and Depression Score [24], HADS, scored 0 to 21 independently for depression and anxiety, with a higher score indicating greater anxiety or depression), previous occupation, and school leaving age. For those who gave informed consent a fasting blood test was taken before breakfast just before the change over and one year later. Blood was tested immediately for haemoglobin levels, and further samples stored at -80°C so that lipids [25,26] from both years were analysed in a single batch.

Anonymous questionnaires including ten questions on enjoyment of food by residents, variety, sufficiency of food and drink, preferred dining area, perceived friendliness of the home and the frequency of visitors sharing meals and drinks were distributed to residents, relatives, and staff before the intervention and a year afterwards. Enjoyment of food was assessed by rating a question 'How much do you enjoy eating here?' from 1 ('I hate eating here') to 5 ('I love eating here'). Questions to relatives and staff asked about residents levels of enjoyment, rather than their own.

The primary outcome for the intervention study was number of falls, as there was a suggested decline in falls in the pilot home. Satisfaction with meals, body weight, dehydration, cognitive functioning, depression, haemoglobin and cholesterol levels were seen as important secondary outcomes.

The changes in food and drink provision, implemented between July and December 2006 in the three intervention homes and detailed in table 1, were intended to improve comfort during meals (making eating more like going to a restaurant than eating in a canteen), increase the level of choice available at meal times, making eating with others a pleasurable and more sociable experience and encourage fading appetites. They were also intended to widen the availability of drinks and snacks (to visitors as well as residents), encourage greater independence on the part of residents in choosing and obtaining their own snacks, and generally reduce the feeling of institutionalisation.

**Table 1:** Description of food and drink provision, and changes

	*Before the intervention and in the control homes*	*After the intervention*
***Breakfast menu***	Choice of porridge, cereal, one type of fruit, fruit juice, toast and marmalade	Choice of cooked breakfast, selection of fruit and fruit juices, cereal and porridge, toast and marmalade

***Lunch menu***	Choice of two different main courses and two desserts	Choice of at least three main courses (including a vegetarian option), soup and salad available, selection of hot and cold desserts, fruit juice

***Evening meal menu***	Choice of two cold options for main course and two desserts	Choice of soup, salad, hot option, selection of sandwiches, selection of desserts

***Timing of choices***	In some homes residents made their lunch choices the day before, others at meal time.	Choice made at the meal time, residents can change their minds even after being given a meal and try something else.

***Display of food***	No food displayed	Cold food displayed as a buffet at the side of the dining-room, hot options can be viewed in a bain-marie

***Environment***	Crowded dining-room, residents use flowery or patterned crockery and table mats	Fewer tables in dining-room, less crowded, refurbished dining-room, tablecloths and flowers on the table, white crockery with side plates for vegetables

***Timing of meals***	Meals at set times (usually 9 am, 12.30 noon and 4:30 pm), single sitting	Dining facilities open for at least one and a half hours at all meal times, several sittings of residents

***Drinks***	Drinks trolley (tea, coffee and evening horlicks) taken round home midmorning, midafternoon and evening	Drinks machines available at all times (hot water for tea, black coffee, cappuccino, hot chocolate, soup) for residents and visitors to use, residents offered drinks by staff midmorning, midafternoon and evening

***Visitors***	Visitors eat at some of the homes on rare occasions	Visitors are welcome to join residents for any meal for a fee

***Snacks***	Biscuits offered with drinks trolley	Selection of biscuits, cakes, savoury nibbles and fresh fruit on display, sandwiches and yoghurts available from a cooler in dining room, all available for self-service at any time

### Analysis

Data from care notes were limited by missing data, as many participants were not present for the whole of the first and second years. To maximise the available data it was decided to analyse each individual's fall rate as falls per month (counting only months for which the participant was living at the home). Poisson regression was used to model the relative rate of falls per person per month after the change for the intervention vs. the control homes, adjusting for the rate of falls before the change, age, use of psychotropic drugs,[27,28] and stratified cluster design using Stata v.10.0 software (each stratum containing a matched pair of homes). Design-adjusted Wald tests were used for calculation of 95% confidence intervals (CI) and P-values for the rate ratios. One resident was omitted from falls analysis as he fell 57 times over nine months, this was an extreme value.

Weight data were more limited as, in addition to censored stays, many homes did not weigh participants during the initial months of the first year despite this being considered good practice, and data were too sparse to compare the change in weight slope over the first year with the second year slope as initially envisaged. For this reason, we calculated, for each individual, the difference between mean weight in year 1 and mean weight in year 2. Adjustment for pair of homes was made through pooling by meta-analysis of weighted mean differences using random effects analysis with each of the 3 pairs entered as an individual study (using RevMan 4.2 software [29]).

Interview data were obtained from fewer participants than care note data. Numbers of participants dehydrated at the second interview were used to calculate a relative risk of dehydration for each pair of homes, and pooled using meta-analysis and random effects methodology on RevMan 4.2 software. MMSE score in year 1 was subtracted from that in year 2 for each participant, and combined data for intervention and control participants compared using the Mann-Whitney test. Depression score in year 1 was subtracted from that in year 2 for each participant and combined data for intervention and control participants compared using the t-test (both analyses without adjustment).

Blood tests were carried out on the smallest group of participants, and only included participants able to give their own informed consent (so that residents of the two dementia care homes were not represented). Only residents with valid samples in both years were included in the analysis. Comparison of mean changes in haemoglobin and total cholesterol levels used a linear regression model (SPSS 14), adjusting for age and the effect of being in a particular pair of homes.

Residents' satisfaction with meals was assessed in questionnaires to residents. As questionnaires were anonymous mean scores for each home in year 1 were subtracted from mean scores at year 2 for that home, and the difference between intervention and control homes analysed using the independent *t*-test.

## Results

**Figure 2:**
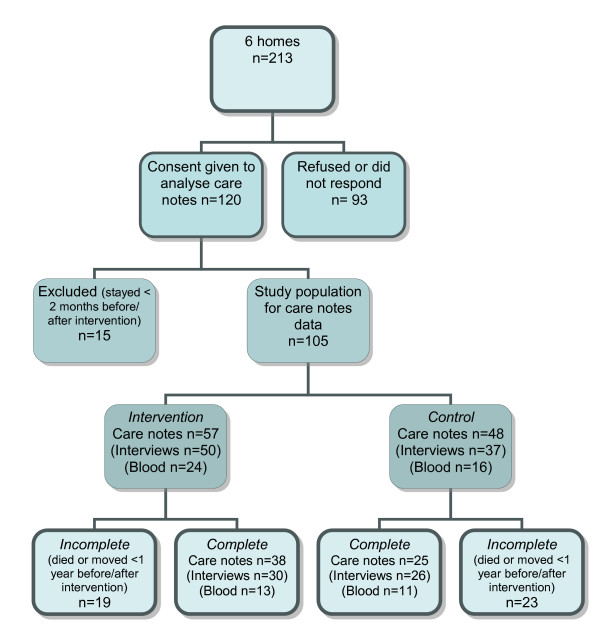
**Study participants flow**.

Of 213 residents in the six homes at the time of recruitment, 120 (56%) residents (or their relatives) agreed to participate in the study (see figure 2). 15 were subsequently excluded, as they stayed living at the home less than two months before or after the intervention point. Thus the study population for data from care notes was 105 residents (57 intervention, 48 control), of which 63 (38 intervention, 25 control) had stayed for the complete study period of one year before and after the change over. Eighty six (49 intervention, 37 control) residents were interviewed, but only 56 participated in interviews in both years (30 intervention, 26 control). 57 blood samples were provided, 22 (13 intervention, 9 control) residents provided blood samples in both years. One in five of the 105 residents who agreed to participate died over the following year.

Questionnaires were distributed anonymously to residents, relatives and staff in both years. 248 (137 intervention, 111 control) responses were received in the first year, and 228 (102 intervention, 126 control) in the second. The total of 476 replies included 200 from residents, 114 from relatives and 162 from staff.

### Characteristics of the population in the first year

The characteristics of the 57 residents in intervention homes and the 48 participants in control homes who gave permission for their care notes to be studied are shown in table 2. Data on other measurements are presented in table 3 and **Additional file 1, tables S1-S2**, and described here briefly. In the first year the residents represented the oldest old (mean age 87) and were predominantly female, with a high risk of health problems. Around 60% of residents had at least one fall during the first year, 40% had at least one wound care visit, and 40% had a urinary tract infection. A high proportion of residents were on diuretics (45%), antidepressants (24%), and psychotropic medication (including anti-depressants, 43%).

**Table 2:** Characteristics of study population, case note data in first and second years*

	First year		Second year	
	**Intervention group**	**Control group**	**Intervention group**	**Control group**

Age, mean (sd)	**86.1 (6.7)**	87.7 (6.8)	**-**	-

Female, n (%)	**38 (67)**	36 (75)	**-**	-

Uses wheelchair, n (%)	**15 (26)**	5 (10)	**-**	-

Diabetic, n (%)	**13 (23)**	2 (4)	**10 (18)**	1 (2)

On anti-depressants, n (%)	**14 (25)**	11 (23)	**-**	-

On psychotropic medication (including anti-depressants), n (%)	**22 (39)**	23 (48)	**-**	-

On diuretics, n (%)	**19 (33)**	28 (58)	**-**	-

Falls/month/resident, mean (sd)	**0.21 (0.32)**	0.12 (0.18)	**0.25 (0.37)**	0.24 (0.35)

Residents not falling during year, n (%)	**23 (40%)**	21 (44%)	**23 (40%)**	24 (50%)

Serious falls (999 dialled), n (%)	**8 (14%)**	3 (6%)	**11 (19%)**	9 (19%)

Number of weight measurements per resident over year, mean (sd)	**4.5 (1.9)**	2.6 (2.2)	**6.5 (2.5)**	4.8 (2.9)

GP contacts per month, mean (sd)	**0.84 (0.84)**	0.54 (0.41)	**0.95 (1.05)**	0.84 (0.94)

Residents with District Nurse wound care contact over year, n (%)	**25 (44%)**	18 (38%)	**22 (39%)**	20 (42%)

Residents with urinary tract infections, n (%)	**21 (37%)**	20 (42%)	**20 (35%)**	12 (25%)

Residents with chest infections, n (%)	**20 (35%)**	10 (21%)	**16 (28%)**	22 (46%)

Residents with hospital stay during year, who survive, n (%)	**13 (28%)**	4 (12%)	**11 (24%)**	7 (21%)

Residents who died during the second year, n (%)	**-**	-	**11 (19%)**	10 (20%)

Residents discharged to a different home during second year, n (%)	**-**	-	**0**	4 (8)

Body weight (kg), mean (sd)	**64.9 (14.68)**	62.31 (12.81)	**64.49 (15.35)**	61.5 (14.57)

Lowest body weight (kg)	**39.0**	40.4	**32.2**	36.1

Highest body weight (kg)	**101.2**	97.1	**101.2**	106.6

Body mass index (BMI), mean (sd), N	**25.75 (5.32) 57**	24.68 (4.49) 43	**25.62 (4.82), 57**	24.3 (4.82), 43

Residents with BMI < 20, n (%)	**6 (11%)**	7(16%)	**8 (14)**	6 (14)

Residents with BMI >30, n (%)	**11 (19%)**	5 (12%)	**13 (23)**	4 (9)

Height (m), mean (sd)	**1.59 (0.09)**	1.58 (0.09)	**-**	-

*For intervention n = 57, control n = 48 (only those with data in both years analysed) unless N specified

**Table 3:** Interview data on cognitive and nutritional status in first and second years*

	First year		Second year	
	**Intervention group**	**Control group**	**Intervention group**	**Control group**

Residents appearing dehydrated, n (%)	**7 (23.2)**	10 (38.5%)	**3 (10%)**	9 (34.6%)

MMSE score (max 30), mean (sd)	**19 (5.6)**	17 (6.2)	**17 (6.2)**	15 (7.9)

Cognitive impairment (MMSE ≤ 23), n (%), N	**25 (83.3)**, **30**	21 (87.5), 24	**22 (81.5)**, **30**	19 (79.2), 24

Upper arm circumference, mean (sd), N	**28.06 (3.06), 30**	27.40 (4.21), 20	**28.9 (3.8), 30**	27.3 (4.97), 20

Grip strength, left hand, mean (sd)	**14.81 (6.37)**	10.72 (4.43)	**13.57 (6.3)**	11.27 (5.92)

Grip strength, right hand, mean (sd)	**11.22 (7.52)**	8.68 (7.34)	**11.72 (6.76)**	11.91 (5.92)

Residents attempting chair stands, n (%)	**6 (20)**	4 (15.4)	**15 (50)**	18 (69.2)

HADS Anxiety score (max 21), mean (sd)	**4.07 (3.00)**	6.3 (4.45)	**4.86 (4.61)**	6.78 (3.83)

Anxious residents (score ≥8), n (%), N	**3 (12%)**, **25**	9 (39%), 43	**4 (16%), 25**	11 (48%), 43

HADS Depression score (of 21), mean (sd)	**5.17 (3.69)**	5.61 (2.98)	**5.04 (3.89)**	6.25 (2.91)

Depressed residents (score ≥8), n (%), N	**7 (28%), 25**	6 (26%), 43	**5 (20%), 25**	7 (30%), 43

* Only those residents who participated in both interviews included, intervention n = 30, control n = 26 except where N is specified.

Nutritional status in year 1 was variable. While mean body mass index (BMI) was 25, 14% were underweight (BMI < 20) and 16% obese (BMI>30). A high proportion of residents appeared dehydrated but this varied widely by home. Residents tended to be very weak, 40% of a small sample of the residents were anaemic, and mean total cholesterol levels were low.

Mean baseline MMSE scores were 18 (of 30), with 85% having some cognitive impairment (score ≤23). Around a quarter of residents were at risk of depression (HADS depression score of 8+) and levels of anxiety were also high.

Generally residents in intervention homes were more positive about their food provision at baseline than those in control homes (although this may have been influenced by knowing that changes in food and drink provision were imminent). Enjoyment of food by residents (as reported by residents) was between 'neutral' (scoring 3) and 'I love eating here' (scoring 5) - with a mean score of 4.1 in intervention homes, 3.5 in control homes. Relative and staff assessments of how much residents enjoyed their meals were less enthusiastic than those of residents in intervention homes, and around the same as residents in control homes.

We compared baseline health and wellbeing of participants living in intervention and control homes in year 1. Residents of intervention homes were slightly younger and less likely to be female. Intervention home residents were more likely to be using a wheelchair, more likely to be diabetic, had higher rates of falls, higher levels of serious falls, higher rates of GP contacts, chest infection, hospital stays and higher levels of obesity. These indications of worse initial health in the intervention homes were countered by lower levels of psychotropic medication use, lower diuretic use, more regular weight checks during the year, lower levels of urinary tract infections, higher mean BMI, lower levels of underweight, lower levels of dehydration, higher levels of cognitive function, greater mid-upper arm circumference, greater grip strength and lower levels of anxiety. Levels of anti-depressant use were similar in residents of both intervention and control homes, as were the proportion of residents falling at least once, and levels of depression. Our summary of this was that there was not a consistent indication of better or worse health and wellbeing in either group, but that there is great variability within these populations, with most participants living with serious health problems.

### Effects of the intervention

#### Falls

Being in a home with the food and drink intervention reduced the rate of falls (falls per person per month) relative to being in a control home by 24% (95% CI -43% to +2%), but this result was not statistically significant (rate ratio 0.76, 95% CI 0.57 to 1.02, p = 0.06). It was adjusted for dissimilarity of falls rates in the first year, gender, use of psychotropic drugs and the effect of the pair of homes. This effect was due to a slight increase in falls from year 1 to year 2 in the intervention homes, and a much bigger increase in falls in the control group (see table 2). The rate of falls in the first year was highly predictive of falls in the second year (as seen elsewhere)[30].

As well as numbers of falls per person we also assessed the numbers of residents falling at least once during the year. In intervention homes 60% of residents fell in both years, while in control homes the percentage of residents falling dropped slightly from 56% to 50%. Numbers of serious falls (where 999 was dialled) rose for both intervention and control homes (from 8 and 3 for incidents in intervention and control homes respectively in the first year to 11 and 9 in the second year). These differences were not statistically significant.

### Nutritional and physical status

On average residents in intervention homes lost 0.4 kg between the first and second years (mean -0.4 kg change, sd 4.7), while residents in the control homes lost more (mean -0.8 kg, sd 5.3). The very high variability reflects that individual residents gained or lost quite large amounts of weight over the two years. Adjusting for clustering there was no significant difference in weight change between intervention and control homes. Compared to controls participants in intervention homes gained 0.63 kg (95% CI -1.2 to +2.4 kg, p = 0.49).

Weight effects in different homes varied- weight change in non-dementia intervention homes was positive (+0.01 kg, sd 5.3 and +0.86 kg, sd 3.6), negative in non-dementia control homes (-0.53 kg, sd 4.1 and -0.50, sd 5.3). In comparison weight loss in both dementia care homes was large (intervention home -2.93 kg, sd 5.0, control home -1.83 kg, sd 5.9). Excluding the dementia care homes data did not suggest a significant positive effect on weight of the intervention (+1.05 kg, 95% CI -0.97 to 3.06, p = 0.31).

Changes in weight of those at the extremes are more important than average changes. It was hoped that the intervention would help to increase the weights of those with lowest body weight at inception, and help to stabilise or decrease weight in those already obese. This was not observed- of the six underweight residents in intervention homes mean weight change was a loss of 2.1 kg, while the 8 underweight residents in control homes gained 0.6 kg on average. The 11 obese residents in intervention homes gained 0.1 kg between the two years, while the 5 obese residents in control homes lost 0.9 kg.

Rates of dehydration dropped in both intervention and control homes at the second interview (16 to 9% vs. 46 to 39% respectively). However, being dehydrated at the first interview did not predict dehydration at the second for individuals. At the second interview the relative risk of being dehydrated in an intervention home compared to a control home was 0.36 (95% CI 0.06 to 2.04, p = 0.25, without taking baseline differences in dehydration between homes into account).

### Cognitive status and mood

Over the year between interviews there was a mean overall reduction in the MMSE score of 2, suggesting a slight deterioration in cognition. Baseline MMSE was slightly higher in intervention than control homes, but both groups dropped in a similar way. This apparent similarity masks differences between residents in different homes. The residents in both of the dementia care homes had the greatest mean decline in MMSE (-2.5 of 30), while residents of one control home increased in their mean MMSE score (+1.0), and residents in its paired intervention home experienced small reductions (-0.5 over the year). Percentages of residents with cognitive impairment dropped in both intervention (83 to 82%) and control homes (88 to 79%) - improvement being more dramatic in the control homes. There was no evidence that the food and drink intervention had an impact on residents cognitive functioning. The Mann-Whitney test showed no significant difference between the change in MMSE score between residents living in intervention and control homes.

A quarter of residents appear to be at risk of depression (HADS depression score of at least 8) in both intervention and control homes and in both years. Some of these residents were on anti-depressants, and some were not. Percentages of residents at risk of depression were reduced in intervention homes (from 28% to 20%) but increased in control homes (from 26% to 30%) from the first to the second interview. Mean change in HADS depression scores from the first to the second interview also reflected a reduction over time in the intervention homes (mean change of -0.16, sd 3.87) and an increase in residents of control homes (mean change +0.57, sd 1.88). There was no significant effect of the food and drink intervention on level of depression (p = 0.42).

### Haemoglobin and lipid levels

Around 40% of residents were anaemic (Hb < 12 g/dl) at the first blood test, rising to around 60% at the second test. Residents' haemoglobin levels were negatively correlated to their age (*r *= -0.431, *p *< .017; older residents had lower haemoglobin levels. Haemoglobin levels increased in intervention homes (mean change +0.03 g/dl, sd 0.98) and decreased in control homes (mean change -0.49 g/dl, sd 0.81) from the first to the second year. Half of this change can be explained by the differences in age between the residents of intervention and control homes. Adjusting for age and effect of home pair, the food and drink intervention has no statistically significant impact on residents' haemoglobin levels.

Total cholesterol and LDL cholesterol levels rose between years one and two in both the intervention homes, while haemoglobin levels in control homes were stable or fell slightly. This was a statistically significant difference between the intervention and control participants, but this significance was lost when data were adjusted for age, as older age correlates with lower total cholesterol. As few participants had blood tests in both years the results in this section were entirely exploratory.

### Enjoyment of food

Residents in all intervention homes, and all but one control home, expressed an increase in enjoyment of meals from year 1 to year 2. While the mean change of residents' perception of their own enjoyment of food and drink in intervention homes (+0.28, sd 0.43) was slightly greater than that in control homes (+0.09, sd 0.63), this difference was not statistically significant (p = 0.237). For details see Additional file 1, table S3.

### Other outcomes

No other outcomes suggested a statistically significant effect of the intervention compared with control once adjustment was made for age and cluster.

### Planning for further studies

This study provides detailed data on levels and variability of many measures in this frail population which may be used to estimate adequate sample size in a future study aiming to assess the effects of this type of intervention on rate of falls or other outcomes. A future study is likely to use a clustered design for implementation of a change in food and drink provision. Sample size calculations for such a clustered design would additionally require an estimate of the intra-class correlation coefficient which measures the ratio of between-home to total (between- plus within-home) variance in the chosen outcomes [31]. If matched pairs of homes are used in a clustered design, as in the current study, then more matched pairs would be required. However, the difficulties of estimating the component of variance in falls that comes from between-home variability from a matched-pair design, and the complex analysis required, may suggest a more relaxed stratified or other variation on the cluster-level design [31]. It would also likely be advisable to adjust analyses for baseline differences in key characteristics which are predictive of the outcome such as baseline fall rate, gender, and use of psychotropic medication.

We could not properly analyse resident's weight changes because in the year before the intervention resident's weights were not being checked very regularly. While Care Quality Commission regulations [32] require that UK care homes assess weight and nutritional status "periodically" this was infrequent in the first year (see table 2). Planning future studies should take into account the dynamic nature of the population of residents, the likely high levels of missing 'routinely collected' data and low levels of consent, especially to taking blood samples.

## Discussion

This study provides a snapshot of the health, wellbeing and nutritional status of 105 Norfolk care home residents. Health problems in this group are very common and research or evaluation studies challenging to conduct - 60% of residents in our sample fell each year, 40% needed wound care, 40% had urinary tract infections and 40% required psychotropic medication. Nutritional problems were also evident, with 15% underweight and 15% obese, around 30% dehydrated at any one time, and 40% anaemic, reflected in low levels of physical strength. A quarter of residents were at risk of depression, a quarter at risk of anxiety and over 80% with significant cognitive impairment. Nevertheless, residents generally declared themselves happy with food and drink provision at their home.

Our pragmatic approach to testing the effect of a service development using available routine data and some additional parameters was partially successful. Of the 105 older people who gave permission for us to access their care notes, 15 were excluded as they were present within the care home for less then two months before or after the change in provision, and a further 19 were not present at the homes for the full two years. Routine data from Norfolk care homes, such as information on falls, GP consultations, weights and outcomes after hospital stays were difficult to gather uniformly because each home has its own way of collecting data, and the data sets are difficult to combine. For example, for many homes it is not clear whether 'GP contact' was a phone call from a member of staff to the GP surgery, or a GP visiting and examining a resident. Similarly, while weight data became more regularly collected for many residents over the period of the study, some had few weights recorded. To conduct a definitive assessment of the effects of changes in food and drink provision in UK care homes, where data collection depends on individual managers and systems, it will be necessary to specifically collect data such as weight, health care contacts and numbers of falls within a research context, rather than relying on routine data collection.

The food and drink intervention tested was associated with a relative reduction in the rate of falls (by about a quarter), but this effect did not quite reach statistical significance. It did not produce statistically significant effects on the number of residents falling, or on secondary outcomes (weight, hydration, anaemia, cognitive status, depression or serum lipids). This may be due to the small sample size, modest effects on our outcomes by the intervention compared to large effects of health problems and increasing frailty, and large variability among residents in the same home and between homes.

### Strengths and Weaknesses

In a small study like this big differences occur between the health and wellbeing of residents of different homes, due to residents' individuality as well as population, geographical, admission and economic differences. On top of this each home has individual management styles, staff, cooks, relatives, friends and volunteers. There were also generic concurrent changes such as nutrition screening (MUST nutritional screening was initiated during this time,[10,33]), a campaign to reduce dehydration introduced across all homes, and introduction of finger foods in both dementia care homes. Furthermore, the system of food and drink provision was already of high quality in many respects at baseline.

This was a pragmatic study - ideally it would have included a greater number of homes, and randomised homes to intervention or control status. But given the County Council's rolling programme of improvements these refinements were not feasible, and we were grateful to be given the freedom and funding to assess the changes in the way that occurred. This commitment to research on the part of the County Council has allowed us to map the complex health risks of this frail older population, begin to understand how such a complex intervention might begin to influence health and wellbeing in this group and provide data to help correctly power a future intervention study.

Use of routinely collected data was of mixed success. While it did provide data that allowed us to assess the health and wellbeing of participants, and effects on participants of the intervention, this was limited by the difficulty of finding the data in various (differing) sets of care notes maintained by the homes and differing levels of detail recorded. We have no way of knowing whether some data were missed due to poor recording or our inability to locate it. The lack of a minimum data set for care homes in the UK make monitoring of the effects of such service improvements, as well as overall standards in care, difficult[34]. Such minimum data sets have been developed for use in the UK, but are not yet in common use[35].

It is possible that residents, staff and relatives knowing about the planned changes (imminently in the intervention homes, one year later in the control homes) may have altered some residences decisions to participate in the study (as the response rate was lower in those in control homes) and may have altered responses to the questionnaires (for which reason the results of the questionnaires have been reported but not over analysed). However, we feel that knowledge of the changes was unlikely to alter falls, or other health and wellbeing outcomes.

### Comparison with other published UK populations

Norfolk residents involved in this study tended to be older than the UK average for care homes (mean age 87, compared to an average age of 86 in women residents and 83 in male residents nationally)[36]. Comparison with other UK institutionalised older populations is restricted due to limited data on other similar UK populations, table 4. Numbers of residents falling, and rates of falling, appear high compared with other UK populations. Levels of cognitive impairment and depression are high [37], but comparable with other UK populations, and it is difficult to draw direct comparisons for GP consultation rates, UTIs, hospital stays and deaths. The improvement in cognitive status seen in one care home has been reported in other populations, and may be due to temporary cognitive impairment in year 1[37].

**Table 4:** Comparison of the Norfolk care home population with other UK older populations

Characteristic	Our Norfolk residents		Other UK population	
	**Numbers**	**Population**	**Numbers**	**Population**

**Rate of falls (per resident, per month)**	0.21 int, 0.12 control (first year), 0.25 and 0.24 (second year)	105 residents of Norfolk care homes	0.19 in control group	3717 residents of 118 British care homes [54]
			0.16 at baseline	661 residents of 13 nursing, 38 residential, 14 mixed homes in Leeds[55]
			0.13 before study began (retrospective data), 0.23 during the 1 year follow up (prospective data)	90 women and 20 men from Cambridge aged 90+ living in the community and in assisted accommodation [30]

**Number of residents with at least one fall**	58% fell over 12 months in first year, 55% in second year.	105 residents of Norfolk care homes	43% fell over 10 months in control group	3717 residents of 118 British care homes [54]
			40% fell over 6 months at baseline, 30% at follow up	661 residents of 13 nursing, 38 residential, 14 mixed homes in Leeds [55]
			59% in 12 months in year 1, 53% in year 2	346 residents of 12 nursing homes with severe dementia in Oxford, London, Newcastle [56]
			58% reported falling in the year before the study, 60% during the years follow up	90 women and 20 men from Cambridge aged 90+ living in the community and in assisted accommodation [30]

**Number with Anaemia**	40% with anaemia in first year, 60% in second year	20 residents of Norfolk care homes	47% with anaemia	4 studies (1481 residents) of nursing homes in developed countries [57]

**Cognitive impairment**	85% of 54 residents (from all 6 homes) had MMSE < 24, or 84% of 37 residents from non-dementia care homes	4 non-"EMI", and 2 dementia care homes.	65% of 445 residents had MMSE < 24	Random sample of residents in 157 "non-EMI" homes in SE England [58]
			23% of 104 had MMSE 0-9, 39% 10-19, 38% 20-30	104 older people admitted to residential homes in Nottingham [59]
			8% MMSE < 24 in non-demented population, 84% < 24 in those with dementia	Over 12,000 randomly selected UK urban and rural participants aged 65+ [60]
	Mean MMSE 19 int, 17 control	4 non-"EMI", and 2 dementia care homes.	Mean MMSE 13.8 and 13.1 in the 2 baseline groups	661 residents of 13 nursing, 38 residential, 14 mixed homes, in Leeds [55]
			Median MMSE 26 (age 80-84) and 25 (age 85+) in men, 25 (80-84) and 24 (85+) in women	Over 12,000 randomly selected UK participants aged 65+ with < 9 years education and without dementia [60]

**Depression**	27% of 48 scored ≥8 on the HADS depression scale	56 residents in 6 Norfolk care homes	38% depressed at admission (reduced over time after admission) 39% depressed at admission (score 5-20 on Geriatric Depression Scale)	188 residents admitted to English care homes who survived >9 months [61] 88 older people admitted to residential homes in Nottingham [59]

**GP consultations, per resident per month**	0.84 int, 0.54 control (including staff phone consultations)	105 residents of residential homes	0.53	661 residents of 13 nursing, 38 residential, 14 mixed homes, in Leeds [55]

**Residents with a hospital stay, %**	28% in int, 12% in control homes over first year, 24% & 21% in second year	105 residents of residential homes	18% over first 6 months, 15% in second six months	661 residents of 13 nursing, 38 residential, 14 mixed homes, in Leeds [55]

**Deaths, % of population over 1 year**	20%	105 residents of residential homes	30%	661 residents of 13 nursing, 38 residential, 14 mixed homes, in Leeds [55]
			30%	346 residents of 12 nursing homes with severe dementia in Oxford, London, Newcastle [56]

EMI: elderly mentally infirm

The suggestion from this small sample, is that the residents of Norfolk residential care homes were slightly heavier and weaker than their counterparts enrolled in the National Diet and Nutrition Survey in 1994/5, table 5[21], in line with general increases in body mass in the UK population over this time, and changes in the criteria used to accept older people into residential care (so that care home populations are becoming frailer).

**Table 5:** Comparison of this population (UK 2006/7) with NDNS data (UK 1994/5) [21]

	Our Norfolk residents	NDNS institutionalised participants
**Age**	86.1 intervention homes, 87.7 control homes	51% aged 85 years and over

**Male**	33% intervention homes, 25% control homes	23%

**Live in residential homes**	100%	57%

**Live in homes managed by local authorities**	100%	21%

**Number of participants giving consent**	105	412

**Consent rates for participation**	57%	94%

**Underweight** **(BMI < 20)**	14%	16% men, 15% women

**Obesity** **(BMI >30)**	16%	10% men, 13% women

**Mean mid-upper arm circumference, cm**	28.4 cm men, 27.5 cm women	26.9 cm men, 27.0 cm women.

**Mean hand grip strength, kg**	16.1 kg men, 9.9 kg women	19.1 kg men, 11.7 kg women.

### Comparison with other intervention studies

A variety of interventions have been tried to improve the nutritional status of older people living in residential care. One study tried flavour enhancement of food over 16 weeks, compared to no intervention in 61 older people living in a nursing home, but found no changes in energy intake or weight[38].

Liquid nutrition supplements are one way to promote nutritional health. One study provided nutrition supplements in the morning to 34 older people with probable Alzheimer's Disease living in a residential setting in a crossover design for 21 days. In those with the lowest bodyweight, who would normally be targeted for nutrition supplementation, there was little effect on energy intake due to reduced lunch consumption following the morning supplements[39]. Another study that provided 250 ml of a liquid nutrition supplement or a placebo to 35 older people with dementia living in a nursing home over 12 weeks found a statistically significant increase in weight in the supplemented vs. the placebo group, but no changes in activities of daily living[40].

Changing the ambience of mealtimes has been tried as an intervention in other studies. Ten wards (178 residents from 5 Dutch nursing homes) were randomised to receive family style meals or the standard service[41]. Standard service was highly institutionalised, and used plastic cups, segmented plates, bibs for residents, choice of meals made two weeks before the event, staff not sitting down but handing out plates then leaving for their own lunch when everyone was served. Cleaning and doctor visits occurred, and drugs were distributed, during the meal, seats were assigned on the basis of availability, meals could not be changed if residents didn't like them and meals began when trays arrived. This was replaced on one ward at each home with a tablecloth, glasses, normal plates, cutlery, napkins, flowers, cooked meal served in dishes on the table, choice of two types of vegetables, meat and carbohydrate foods, at least one member of staff, assistant or volunteer per table, sitting down and chatting with residents, drugs handed out before the meal, most residents serving themselves, eating when all residents were sitting down, and no other activities occurring during the meal. This dramatic change in style resulted in improved quality of life, improved physical function and improved body weight in the intervention residents compared with control residents over six months. A similar, but smaller and earlier study did not show statistically significant effects[42]. It may be that the larger Dutch study achieved statistically significant effects because the levels of resident socialising and control at baseline were so very low - whilst important changes were made in Norfolk they were not as dramatic as in the Netherlands due to much more appropriate levels of care during meals during the first year.

One small US study replaced plated meals at the evening meal with a buffet-style choice in 40 older residents of a long term unit and with enhancement of the dining environment (tablecloths, china, decorations, residents positioned for social engagement). In this study there were no significant differences between the intervention and control participants in weight or haemoglobin levels[43]. A before-after study assessed the effect of portioning food in the residents dining room (as compared to pre-plated meals) on 34 older residents with dementia over 3 months. They found no statistically significant effects on weight, BMI, mid-upper arm circumference or haemoglobin, although intake appeared to rise[44]. Another small study found greater energy intake with a new bulk delivery system (including a new unit with enhanced dining atmosphere), compared to earlier tray delivery, in 49 cognitively impaired older residents, and weight gain was greater in those with low BMI at start[45]. The literature on feeding people with dementia has been systematically reviewed, finding that there are few studies, with weak methodology and small sample sizes to inform interventions to improve feeding for those with dementia[46]. There are ongoing cohort studies addressing the effects of setting[47,48].

We chose falls as our primary outcome for several reasons. Falls are an important component of morbidity and mortality in frail older people, and falls are the result of a great many factors which can combine to increase risk. Many of the factors that we hoped to influence through the food and drink intervention, such as dehydration, muscle strength, anaemia, depression, cognitive status (and medications affecting depression and cognition) and undernutrition could all impact on falls[49-51]. In addition a reduction in emotional stress within the home as a result of increased relaxation around social eating could help to protect older people [52] though it may be that this sort of structural change in combination with individual assessment and intervention may be most effective[53].

## Conclusions

Care home residents are frail and experience multiple health risks. This intervention to improve food and drink provision was well received by residents, and a 24% relative reduction in falls was observed (due to a greater rise in falls between year 1 and 2 in control homes than in intervention homes), although in this small and varied sample statistical significance was not attained (p = 0.06). While this study was not ideal in design or power we hope that, given the paucity of published interventions to improve the atmosphere for residents dining in care homes, it will help to inform best practice in the area and provide data to help in planning of further research in this challenging context. Further research is needed to understand which, if any, components of the intervention may be successful in improving the health and wellbeing of older people living in residential care.

## Competing interests

The research was funded by Norfolk County Council, and JB works for Norfolk County Council. These links did not affect the way that the data are presented.

## Authors' contributions

**AK **collected much of the quantitative data presented in this paper, collated and analysed the data, and drew up the tables and figures and fed back the findings to stakeholders. **GP **acted as statistical advisor to the project, and analysed the falls data. **JB **worked with the meals project committee to devise the intervention, developed the risk assessment, and managed the home that first switched its food and drink provision as part of this trial. **LH **was PI, devised the assessment, applied for ethical approval, supervised data collection, wrote the first draft of the manuscript, is the guarantor and is corresponding author. All authors read and commented on the manuscript before publication and approved the final manuscript.

## Pre-publication history

The pre-publication history for this paper can be accessed here:

http://www.biomedcentral.com/1471-2318/10/28/prepub
